# The effects of graduate competency-based education and mastery learning on patient care and return on investment: a narrative review of basic anesthetic procedures

**DOI:** 10.1186/s12909-018-1262-7

**Published:** 2018-06-28

**Authors:** Claus Hedebo Bisgaard, Sune Leisgaard Mørck Rubak, Svein Aage Rodt, Jens Aage Kølsen Petersen, Peter Musaeus

**Affiliations:** 1 Centre for Health Sciences Education, Faculty of Health, Aarhus University, Palle Juul Jensens Boulevard 82, Building B, DK-8200 Aarhus N, Denmark; 2 Department of Paediatrics and Adolescent Medicine, Aarhus University Hospital, Palle Juul Jensens Boulevard 99, DK-8200 Aarhus N, Denmark; 3 Department of Anaesthesiology and Intensive Care, South Section, Aarhus University Hospital, Tage-Hansens Gade 2, 8000 Aarhus C, Denmark; 4 Department of Anesthesiology and Intensive Care, North Section, Aarhus University Hospital, Nørrebrogade 44, 8000 Aarhus C, Denmark

**Keywords:** Anesthesia, Graduate medical education, Internship and residency, Catheterization, Central venous, Anesthesia, General, Anesthesia spinal, Anesthesia, Epidural, Airway management, Competency-based education, Mastery learning

## Abstract

**Background:**

Despite the widespread implementation of competency-based education, evidence of ensuing enhanced patient care and cost-benefit remains scarce. This narrative review uses the Kirkpatrick/Phillips model to investigate the patient-related and organizational effects of graduate competency-based medical education for five basic anesthetic procedures.

**Methods:**

The MEDLINE, ERIC, CINAHL, and Embase databases were searched for papers reporting results in Kirkpatrick/Phillips levels 3–5 from graduate competency-based education for five basic anesthetic procedures. A gray literature search was conducted by reference search in Google Scholar.

**Results:**

In all, 38 studies were included, predominantly concerning central venous catheterization. Three studies reported significant cost-effectiveness by reducing infection rates for central venous catheterization. Furthermore, the procedural competency, retention of skills and patient care as evaluated by fewer complications improved in 20 of the reported studies.

**Conclusion:**

Evidence suggests that competency-based education with procedural central venous catheterization courses have positive effects on patient care and are both cost-effective. However, more rigorously controlled and reproducible studies are needed. Specifically, future studies could focus on organizational effects and the possibility of transferability to other medical specialties and the broader healthcare system.

## Background

During the past two decades, medical educators and regulators have introduced competency-based education (CBE) and mastery learning (ML) into the graduate medical curriculum in many specialties including anesthesiology [1, 2]. This narrative review evaluates the patient-related and cost-benefit outcomes in CBE-literature for five basic anesthetic procedures.

### Competency-based education

The origins of CBE can be traced to outcomes-based education in the 1950’s, based on behavioristic learning theory [3]. In this theory, the trainees are seen as impressionable to outside influences that create learning outcomes regardless of innate capabilities or processes. Outcomes here are conceived as observable behavioral changes in the trainees following training. This outcome, defined by experts in the field of CBE, is called competence [3]. The duration of training before reaching competence is individual and is a result of both the learner’s aptitude and the teaching offered [4]. The variable educational time necessary to reach the fixed outcome of competence is in contrast to the traditional fixed duration of curriculum concluding with a variable outcome assessed by grades [5]. CBE-based courses thus focus on the eventual outcome of the education rather than on the educational methods and duration [6].

Mastery learning can be conceived as a more rigid form of CBE. In ML, a high level of mastery, originally defined as 90% correct answers, is needed for the learner to progress to a more advanced level of training or to be asserted as proficient [7]. Educationalists such as Keller, Carroll and Bloom proposed that up to 90% of all learners could reach mastery level if offered the appropriate educational method and the right time for learning the subject [4, 8, 9]. Continuous formative evaluation of learning is necessary for the trainee to reach mastery, identifying parts still needed for remedial teaching before the desired level of mastery is achieved. [10].

### CBE in medical education

Introduced into medical education by McGaghie and colleagues with their World Health Organization paper in 1978 [11], CBE has, particularly since the late 1990’s, seen a rapid international growth, dissemination, and adaptation [12]. Large educational governing bodies such as the Accreditation Council for Graduate Medical Education of the USA [13] and Royal College of Physicians and Surgeons of Canada [14] have created overarching competence frameworks to assist in the design and implementation of CBE. These and related frameworks have been implemented in several specialties, among these anesthesiology specialty training programs in the USA, in the UK and in Continental Europe including Denmark [15–24].

One of the driving forces behind the shift to CBE was the reduced work hours for trainee doctors introduced by governing bodies internationally [25, 26]. The reduced work hours were thought to decrease the exposure to cases upon which graduate medical education traditionally relied in a fixed-duration training program [1, 27, 28]. CBE and ML are seen as means of enabling a more systematic acquisition of skills, which mitigates the effect of reducing work hours [29]. Specialist accreditation was traditionally awarded by completing the fixed-duration training and by written knowledge tests [30]. CBE and ML are thought to provide more transparent and relevant clinical outcome measures for assessment of specialist accreditation [31].

### Criticism of CBE

Although CBE seems to answer the aforementioned problematic work hour restraint in graduate medical education, it has seen opposition as well. As a result of the enthusiasm it is experiencing, CBE in graduate medical education is criticized for infallibility, deeming conceptual criticism as invalid [32]. CBE is further thought to atomize the complex field of medical expertise into checklists, concerning itself with subsets of skills or discrete tasks, all the while only evaluating to minimum standards [33–35]. According to critics, the complex order of proficiency or expertise is not directly observable, and CBE thus risks ignoring the time and experience needed to form proficiency and medical expertise [35–38]. Critique of CBE and ML is further concerned with the potentially increased costs due to enhanced supervision, education of supervisors and the variable duration of training [36, 37, 39].

Considering the time and funding already invested in clinical training [40–45], it is thus relevant to examine whether skills training by CBE and ML transfers into clinical performance and patient care and delivers a return on investment. Indeed, recent reviews emphasize the need for further research to qualify the effects and identify tangible therapeutic and organizational outcomes [46–52]. An appropriate model of evaluation is necessary to answer this question.

### The Kirkpatrick/Phillips model for training evaluation

The original Kirkpatrick model has four sequential levels: reaction, learning, behavior, and results [53]. Positive results at a lower level are necessary for causal inference of a superior level effect as the result of an education intervention [54]. Phillips added a fifth level, “return on investment”, to the four original levels [55]. This fifth level evaluates the trade-off between the costs of the training program and the revenues created by the effects of the program. The costs of the program can be the investment in and maintenance of equipment and salary for the trainers and trainees. Revenues could be decreased complications, shorter hospital stays and added contributions to department clinical services [55].

**Fig. 1:**
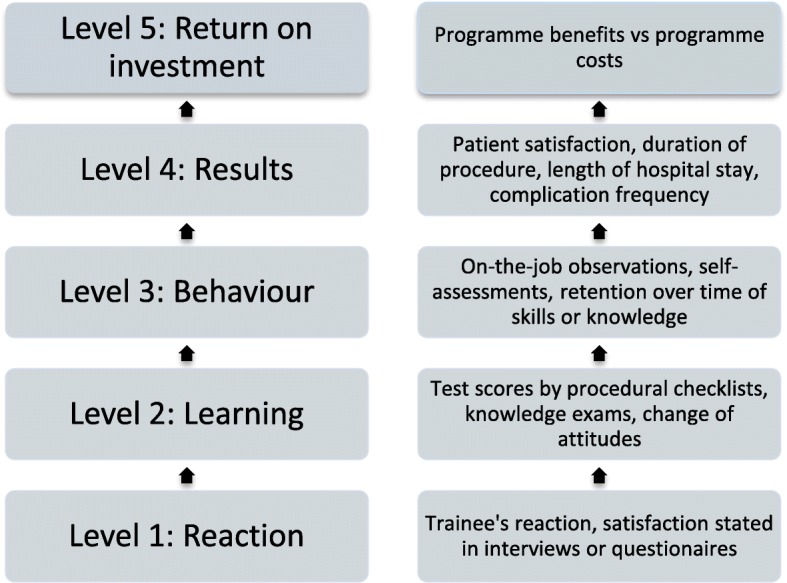
Kirkpatrick/Phillips, adapted to medical education

An adaptation of the Kirkpatrick model to medical education has been proposed by Bewley [56]. This model, with the addition of retention as a measure of sustainable behavioral change over time, is used as inspiration for this review. The resulting adaptation to the Kirkpatrick/Phillips model for training evaluation is presented in Fig. 1.

The appeal of the Kirkpatrick model is the simplicity it proposes to an otherwise complex framework of influences by categorizing the outcome in four categories. The model emphasizes level 4, results, as the most important outcome level that an organization can readily assess if the training adds value. In the case of graduate medical education, level 4 would concern patient care [57]. Furthermore, the Kirkpatrick/Phillips model is also widely used in medical education and is thus readily recognizable to readers [49, 57].

The weakness of the Kirkpatrick model is closely related to its strength. The focus on outcomes risks omitting the focus on the process of learning. In addition, the automatic causality inference often implied in Kirkpatrick analyses is seen as overly simplistic [54]. Many influences other than the training intervention itself can contribute to enhanced results, as exemplified by the Hawthorne effect [58]. Here, the mere extra focus on the subjects of the investigation, rather than the intended intervention, is thought to have produced results.

The Kirkpatrick/Phillips model was chosen for this review as a recognizable framework of clearly defined levels. Using the Kirkpatrick framework as intended, our outcomes should be defined. The outcome of medical education should be the competent physician best suited for the patients’ and society’s needs [11]. This translates into the competent performance of skills in the treatment of patients, which ultimately leads to enhanced patient care. Training should additionally be cost-effective in order to justify the training expenditures [59]. These criteria translate into effects evaluated by Kirkpatrick/Phillips levels 3–5.

### Study aim

1. The current narrative review assesses the literature on outcomes pertaining Kirkpatrick/Phillips Levels 3–5 (clinical skills performance, patient care and return on investment) originating from CBE or ML training for five basic anesthetic competences: airway management, spinal anesthesia, epidural anesthesia, and central venous catheterization.
2. Furthermore, this review will identify gaps in the literature and discuss the implications for training that could be drawn from this discussion.
3. Finally, future directions for enhancing the evidence will be evaluated.

## Methods

A narrative review, able to encompass a large heterogeneity of studies, was chosen as the best method for the present study for several reasons [60–62]. First, the field of CBE and ML is broad, covering research from both traditional simulation and workplace learning. A narrative review would enable the evaluations of the evidence, gaps and future directions. Second, the empirical studies encompass different study designs and varied quality in terms of design and measurement. In light of these two characteristics of the literature, we decided to perform a narrative overview of the subject instead of attempting to calculate aggregated effects in a systematic review.

The narrative review was conducted by first defining the searchable keywords by the PICO framework (population, intervention, control, outcome) [63]. The PICO framework is a mnemonic used to break a research question or aim into searchable keywords by categorizing them into four items [64]:

- **Population**: Residents or interns involved in graduate procedural training.
- **Intervention**: Mastery learning or competency-based training courses of the procedures of general anesthesia, airway management, spinal anesthesia, epidural anesthesia/analgesia, and central venous catheterization.
- **Control**: Other intervention, normal or traditional training or none.
- **Outcome**: Reporting a level 3 or superior outcome, including retention over time, according to the Bewley adaptation of the Kirkpatrick/Phillips model for training evaluation.

### Data sources

A search was conducted using the MEDLINE, ERIC, CINAHL and Embase databases. The search was for English language literature on medical education and anesthesiology literature from January 1946 to August 2017.

Google Scholar was searched for gray literature by reviewing both references included in and papers citing the selected studies from the primary search [65].

### Search strategy

For the primary MEDLINE search, the MeSH terms and Boolean operators “education, medical, graduate” OR “internship and residency” were applied. The search results were subsequently narrowed by combining these terms with the MeSH terms concerning the relevant procedural keywords: “catheterization, central venous” OR “anesthesia, epidural” OR “analgesia, epidural” OR “anesthesia, general” OR “airway management” OR “anesthesia, spinal”.

A similar search strategy was conducted in EMBASE.

CINAHL was broadly searched for the words “competency-based education” or “mastery learning” coupled with the procedural keywords. ERIC was searched broadly for the procedural keywords only.

### Selection of papers

The MEDLINE, ERIC, CINAHL and Embase databases were searched. The first author read the titles and abstracts for adherence to the inclusion criteria:

- English language
- CBE and ML-training interventions, either declared or undeclared, but in design
- Studies concerning postgraduate medical training on resident or intern level
- Studies reporting results concerning Kirkpatrick-Phillips levels 3–5, including retention of skills over time.

Published from January 1946 to August 2017.

The following exclusion criteria were applied:

- Non-CBE and non-ML interventions
- Studies only reporting immediate skills acquisition in a simulated setting
- Studies reporting the training of medical students, nurses, attending, fellows or specialists.

The author group subsequently read the resulting selection of studies in depth for adherence to the inclusion criteria. From this primary selection, Google Scholar was used to search for references in the papers and papers referring to the primary selected papers [65].

**Fig. 2:**
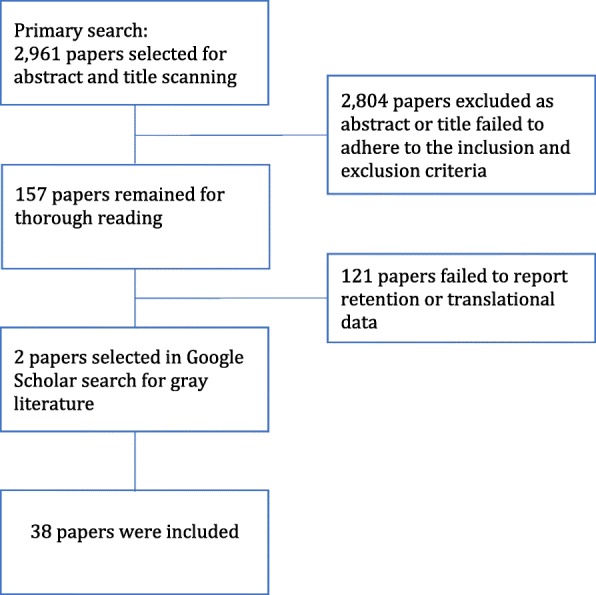
Selection Process

The search strategy and the resulting number of papers are shown in Fig. 2: Selection flowchart.

The author group read the final selection of papers in depth. Data on competency type, intervention training type, duration and number of intervention group trainees were extracted from the papers. Furthermore, data on control group type and training were recorded. Finally, the study outcomes were registered and categorized according to the Kirkpatrick/Phillips model.

## Results

The 38 papers selected for review are shown in Table 1: Selected Studies.

**Table 1:** Selected studies

Paper Procedure Kirkpatrick	Intervention training Duration Number (N)	Control training Allocation of controls Number (N)	Principal findings	Comments and reflections
Level 5				
Burden [66] CVC Level 4, 5	ML: Didactic lecture and simulation practice with feedback 4 h *N* =?	Insertion in patients supervised by senior staff Historical controls *N* =?	Annual savings from decrease in infection $540,000	Cost-benefit from actual financial data adds strength to conclusion. CBE as part of bundle, pre-post CBE-setup. Other interventions than only ML-course
Cohen [67] CVC Level 4, 5	ML: Lecture and simulation training with feedback 4 h *N* = 69	Traditionally trained, five supervised insertions Historical controls *N* =?	Annual savings from decrease in infection $700,000	Even including one-time investments, still resulted in positive Cost-benefit
Sherertz [68] CVC Level 3, 4, 5	CBE: Lecture, series of hands-on stations, one CVC. 3 h *N* = 406	Conventional bedside and didactic instruction Historical controls *N* = 140	Cost savings from decrease in infection 63,000-$800,000	CVC Infection control course, large groups, other relevant procedures taught
Level 4				
Evans [76] CVC Level 3, 4	ML: Added lecture, video presentations, observed simulated hands-on 1–8 attempts *N* = 90	“see one, do one, teach one.” Five supervised insertions on patients Concurrent, randomized controls *N* = 95	Significantly higher first pass success rate in clinical setting	Ultra sound guided, Very low complications, pre and post-intervention
Smith [81] CVC Level 3, 4	CBE: Added case based didactic discussion, hands on simulation training 2 h *N* = 25(20)	Supervised performance on patients with immediate feedback Concurrent, randomized controls *N* = 27(8)	Intervention significant better knowledge and comfort in post-test, no difference to controls at 3 months. No difference in complications, nor needle passes	Skills decline over 3 months as argument for renewed skills training.
Khouli [69] CVC Level 4	CBE: Video and debriefing of hands on simulation training ? duration *N* = 24	Historical: Apprenticeship model “see one, do one, teach one.” Concurrent: Video only Historical and Concurrent, randomized controls *N* = 23	Significantly lower infection rate in interventional department than in the control group and historically	Strength from RCT-setup and well-defined control group training. Comparison to other enhanced training to account for Hawthorne effect
Miranda [93] CVC Level 3, 4	CBE: Presentation, observed and supervised hands on simulated training 2.5 h *N* = 40	Usual ward orientation Firm based allocation *N* = 110	Significantly larger increase in knowledge in intervention group, no difference in success rate.	No change in behavior or patient care, despite practical intervention. Infrequent insertion rate perhaps responsible for non-sustainable results.
Udani [77] Spinal anesthesia Level 3, 4	ML: additional training with deliberate practice and immediate feedback ? duration *N* = 10	Base curriculum of written teaching materials and 15-min video Randomized, concurrent controls *N* = 11	Significantly better checklist scores post-training, higher failure rate in intervention group	Randomized and well-described control group training. Immediate transfer of simulated best clinical practice skills to real patients, enhances patient safety in early procedural training
Britt [70] CVC 3, 4	ML: additional hand-on demonstration and performance ? duration *N* = 13	Standard lecture followed by supervised training on patients Randomized, concurrent controls *N* = 21	Nonsignificant lower complication rate in intervention group, no effect on infection rate	Randomized. Just short of statistical significance for level 4-measures, population too small.
Barsuk [78] CVC Level 3, 4	ML: lecture, ultrasound and simulator training with feedback 4–5 h *N* = 28	Traditionally trained, five supervised insertions Historical controls *N* = 13	Intervention group needed fewer needle passes in clinical performance	Only self-reported data on needle passes and self-confidence, introduces possible reporting bias.
Barsuk [71] CVC Level 4	ML: lecture, ultrasound and simulator training with feedback 4–5 h *N* = 92	Lecture series, no formal training Historical controls in same ICU and concurrent controls in other ICU *N* =?	Significantly lower infection rate in intervention group compared with historically and control group	Historical controls, no control for Hawthorne effect of altered behavior not stemming from the practical hands-on training.
Peltan [94] CVC Level 3, 4	ML: added supervised practice on simulator 1–2 h additional *N* = 36	Lecture, interactive online module, familiarization to CVC equipment, instruction at all procedures Randomized, concurrent controls *N* = 37	Significant improvement in adherence to procedural protocol, no difference in other clinical performance	Strength from randomization. Direct observation enhances reliability of results. Equal clinical performance raises questions of the appropriateness of procedural protocol for improving outcome.
Barsuk [72] CVC Level 3, 4	ML: lecture, ultrasound and simulator training with feedback 4–5 h *N* = 51	Lectures and by observing more experienced physicians performing CVC Historical controls *N* =?	Significant decrease in infection rate post-intervention in different hospital setting.	Enhances generalizability for results of the intervention, highlights the effort needed for implementation to succeed and the vulnerability of the intervention.
Sekiguchi [73] CVC Level 3, 4	CBE: Interactive video, hands on training 105 min *N* = 56	Supervision of 10 subclavian, 10 internal jugular and 5 femoral vein insertions or 10 ultrasound guided procedures Historical controls *N* =?	Significant post-interventional decrease in complications, interns as fellows and attending physicians	Coinciding with increase in Ultrasound Guided insertion, which in itself decreases risk of mechanical error, possible confounder.
Hoskote [74] CVC Level 4	CBE: Debriefing on simulated pre-test, hands on training and repetitive simulated practice ? duration *N* = 60	Not stated Historical controls N =?	Change in policy owing to decrease in infection rate to benchmark level	Good example of organizational change due to enhances in procedural safety following CBE training program
Koh [83] CVC Level 3, 4	CBE: lecture, video demonstration, simulation ? duration, 5 CVCs *N* = 32	No controls	Learning curve of 7 CVCs performed before acceptable complication and success rate reached	Not directly related to the training course, but interesting to establish learning curve
Martin [75] CVC Level 3, 4	CBE: Didactic sessions, supervised skills training on cadaver, videotaped and reviewed for repeated sessions ? duration *N* =?	Advanced cardiac life support and advanced trauma and life support courses Historical controls *N* =?	Significant decrease in pneumothorax at 3 months, non-significant at 1 year	Although pre-graduate intervention, the results are postgraduate. Argument for effect of early training despite many procedures trained at the same time
Smith [109] Fiberoptic intubation Level 4	ML: Written instruction, simulated then clinical supervised training Min 1 h *N* = 12	No controls	95% completed within benchmark duration	Learning curve interesting for expected skills development in training.
Barsuk [99] CVC Level 4	ML: lecture, ultrasound and simulator training with feedback 0 h *N* = 102	Lecture series, no formal training Historical controls *N* =?	Trickle-down effect of pre-test increase after first years of mastery learning course	Potential for additional effect of a training program, what kind of learning is transferred passively.
Level 3				
Friedman [95] EDC Level 3	CBE: Lectures on EDC insertion. High fidelity EDC-model 60 min, 15 insertions *N* = 12	CBE: Lectures on EDC insertion. Low-fidelity banana model Randomized, concurrent controls *N* = 12	No difference between hi- and lo-fi intervention, higher score by experience	Effects from inexpensive models comparable to more expensive could reduce costs of training, leading to higher cost-benefit
Scavone [80] General Anesthesia Level 3	CBE: General anesthesia for emergency cesarean delivery ? duration *N* =?	Lecture and General anesthesia scenario, unrelated to obstetric emergency Randomized, concurrent controls *N* =?	No difference in time to incision or confidence, I-group better score in repeated simulation	Adherence to scoring system perhaps enhanced safety, but did not lead to earlier operation, which would be a desirable outcome in real world.
Gaies [85] Bag mask ventilation Level 3	CBE: didactic session, observation and hands on, supervised practice ? duration *N* = 18	Observing more experienced clinicians Block randomized controls *N* = 20	Significant skills decline in both groups in final test	Rapid decline in skills after early skills training, rarely performed procedure
Kulcsar [98] Spinal Anesthesia Level 3	CBE: Same teaching, but by simulator with haptic feedback 110 min *N* = 14	Practical procedural subparts teaching, using an orange Randomized, concurrent controls *N* = 13	Non-significantly increased scores on clinical performance.	Less than half were tested clinically, very small study groups. Short follow-up 3 weeks.
Barsuk [96] CVC Level 3	ML: lecture, ultrasound and simulator training with feedback 4–5 h *N* = 76	Lecture series, no formal training Historical Controls *N* = 27	No difference in quality indicators in clinical performance between groups	Only self-reported data of complications, risk of reporting bias in the intervention group.
Chan [101] CVC Level 3	CBE: Instruction and demonstration in parts, followed by practice 61/50 min average *N* = 11 (part)	Instruction and demonstration in whole procedure, followed by practice Randomized, concurrent controls *N* = 8	Only Part Task significant better in Global Rating Scale at one-month retention, rest no difference.	Interesting that the difference was found in the overall global rating scale and not in the check lists for parts of the procedure, when comparing whole to part-task instruction
Friedman [79] EDC Level 3	CBE: Added 17 min demonstration video on aseptic technique 75 min *N* = 18	Lecture on aseptic technique Historical controls *N* = 11	Significantly better scores at all intervals and in overall score of skills retention	Unclear to the extent of difference in training, only a new video or the subsequent clinical supervision as well?
Ortner [84] General Anesthesia Level 3	CBE: Full-scale general anesthesia scenario, supervised and debriefed ? duration *N* = 24	Traditionally trained attending physicians as benchmark performers *N* = 6	Trainees reached benchmark level of attending physicians immediately and at 8 months	The short course seems as effective as experience in sustainable skills for a multidisciplinary procedure
Finan [97] Airway management Level 3	CBE: Didactic component, demonstration and supervised hands-on simulator training 2 h *N* = 13	Standard course, one of more skills training sessions and subsequent clinical experience Historical controls *N* =?	Significant lower clinical success rate and return to baseline skills after immediate effect	Kirkpatrick level 1 and 2 reached but could not be transferred into clinical practice. Cause? Fidelity, simulation not encompassing the variability of real life?
Millington [82] CVC Level 3	CBE: Multimedia educational material, demonstration followed by hands-on training 2 h *N* = 30, 16 in retention phase	No controls	Significant increase in retention of knowledge, immediate post-training increase in skills also	Retention test of skills would have been preferable to knowledge retention as an effect measure since other studies have shown retention discrepancies between the two.
Garood [86] CVC Level 3	CBE: One of more skills training stations in one day course. Small group training One day *N* = 41	No controls	Immediate confidence increase, significant decrease at 3 months	Self-reported confidence in clinical encounters is a weak measure of learning effect, subject to reporting bias.
Lenchus [100] CVC Level 3	CBE: Video instruction, discussion, instruction on ultrasound guidance, demonstration, individual practice 160 min *N* = 60	No controls	Significant improvement in clinical performance score	Very short training time, but until competency? Better adherence to checklist = better procedural performance or clinical outcome.
Lenchus [103] CVC Level 3	CBE: Video instruction, discussion, instruction on ultrasound guidance, demonstration, individual practice 4 h *N* = 60	No controls	Significant immediate improvement in knowledge and procedural checklist score	Same setting as above, unclear if the post-instruction score was on the first real patient performance.
Thomas [87] CVC Level 3	ML: Instructional video, supervised hands-on training 60–90 min *N* = 26	No controls	Confidence significantly improved at 3 months, clinical scores deteriorated.	Argument for mandatory retesting and training, as residents own perception of skills was incongruent with actual skills performance after three months.
Barsuk [88] CVC Level 3	ML: lecture, ultrasound and simulator training with feedback 4 h *N* = 49	No controls	Significant decline in skills test at 6 and 12 months after initial improvement.	Another powerful argument for repeated testing and remedial training, skills decay over time if not.
Laack [89] CVC Level 3	ML: interactive learning stations of part tasks, supervised 4 h *N* = 26	No controls	Significant skill decay after 3 months	Remedial training argument for maintenance of initially acquired skills
Siddiqui [90] EDC Level 3	ML: Lecture, video, hands-on training on lo-fi model Duration > 45 min *N* = 21	No controls	Retention score consistently over benchmark	Strong argument for hands-on training, also for aseptic technique
Diederich [91] CVC Level 3	ML: Low-fidelity mannequin trainer, instructional video, partwise instruction and hands-on training with immediate feedback ? duration *N* = 20 (Low-fidelity)	ML: High-fidelity mannequin trainer, instructional video, partwise instruction and hands-on training with immediate feedback *N* = 20 (High-fidelity)	Both groups performed above the minimum passing score at 4 weeks retention test	Strength from randomization and from well-defined ML-interventions in both groups. Possible cost-saving potential from low-fi non-inferiority. Short follow-up (4 weeks).
Cartier [92] CVC Level 3	CBE: Instructional video and hands-on, videotaped simulations, supervised by peers ? duration *N* = 37, 18 sustainability tested	No controls	Significant skills and knowledge increase from pre-training to post training and subsequent decline to > 2 years sustainability test.	Possible Hawthorne-effect from one cohort pre-post testing. Large dropout to sustainability. Still effect of training although diminishing after 2 years as argument for remedial training at interval shorter intervals than 2 years.

? = Unknown

Three papers reported results on Phillips Level 5, return on investment [66–68], all concerned with central venous catheterization. All three demonstrated a return on investment from a novel CBE-training program for CVC-insertion because of a decrease in complications and the related costs.

Eighteen studies showed effects of level 4: learning. They investigated competence in CVC (16 papers), spinal block (1), and airway management (1). For the CVC studies, 9 papers reported rates of complications [66, 68–75], and 3 papers reported needle passes and success rates as predictors of complications [76–78].

A total of 31 papers reported results concerning Kirkpatrick level 3: behavior. CVC was the predominant procedure (22 papers), followed by general anesthesia and airway management (4), epidural (3), and spinal anesthesia (2). Retention of skills was reported in 16 studies [68, 76, 79–92].

## Discussion

### Primary findings

The results of all three studies investigating Kirkpatrick Level 5 show cost-saving potential because of prevention of complications and improved patient outcomes. The studies report return on investment in the range of a minimum of $63,000 over 18 months up to $700,000 per year [66–68], thus creating a strong argument for the investment in CBE for CVC-training.

In addition to the studies reporting return on investment, six studies show that CBE-training courses benefit patient care by significantly diminishing the complication rate in CVC placement [66–69, 71–75]. Furthermore, two studies demonstrate significantly fewer needle passes as a strong measure for decreased complications risk [76, 78]**.** Although four studies show no difference [70, 81, 93, 94] and one a negative effect on success rate [77], these results indicate a positive effect on patient care from CBE-trained CVC insertion.

Twelve of the reviewed studies for level 3 fail to find lasting effects [81, 82, 85–89, 92, 95–97]**,** and six struggle to find an initial effect for the immediate skill transfer to patient care [70, 80, 93, 95, 96, 98]. This contrast to the predominantly positive results from levels 5 and, in part, 4 is interesting. The reason for this contrast to higher-level studies could be that non-effective lower-level studies would not lead to the research of higher-level effects, due to the sequential nature of Kirkpatrick’s model [53].

### Detailed findings

#### Kirkpatrick levels

The causality of Kirkpatrick higher-level learning outcomes warrants precaution if learning outcomes at lower levels have not been evaluated sufficiently [57]. It is thus preferable to demonstrate effects from training at the lower levels before attempting to prove higher-level gains [53, 55].

Of the three studies reporting level 5 results, the studies by Cohen and Sherertz satisfies this requirement of sequential training evaluation. Sherertz in the same study evaluates trainees’ satisfaction, change in clinical behavior, and the ensuing decrease in complication rate, which leads to the economic return on investment [68]. Cohen [67] inherits the sequential effects of the lower-level effects from investigating the same intervention in previous studies.

The studies from Cohen, Barsuk and coworkers are textbook examples of the stepwise evaluation of an educational intervention accommodating the Kirkpatrick principle [67, 71, 78, 88, 96]. The studies of the same intervention have established results from clinical performance and retention of skills by score cards, a decrease in the number of complications, and ultimately the positive return on investment in the Cohen paper [67]. The likelihood of the educational intervention being the cause of the higher-level effects therefore increases.

The dissemination study from Barsuk [72] shows that the same educational intervention can be transferred to a different hospital setting and still leads to improved patient safety and outcome at Kirkpatrick/Phillips level 4. Coupled with the trickle-down effect of the Barsuk 2011 study [99], it adds to the impression of a generalizable positive effect from the studied intervention. In this study, trainees showed improved pretraining procedural scores by simply observing their more experienced colleagues, who had already completed the program. This effect infers the possibility of raising the expected mastery level without adding cost, thus adding to the already established return on investment of the study by Cohen et al. [67].

#### Educational strategy

The investigated training courses are predominantly lectures and hands-on training of 45 min [90] to five hours [88] before allowing for clinical procedural performance on patients, either supervised or unsupervised. As critics have noted, these relatively short courses carry the risk of training for minimum requirements [33, 35].

When continued supervision in the clinical setting occurs, the supervisor can assist with further procedural instruction, which might enhance the procedural proficiency before independent performance. In the trial setting, the added clinical training represents a potential bias if differences in supervision between subjects are present.

In the unsupervised clinical performance, further development of skills is left to the trainees’ own practice. The expected competence of the training course should thus be well defined and ensure a safe performance of the procedure following the course in order to minimize patient risk of complications.

Unfortunately, the transparency of competency level in the included CBE studies is not always as clear as CBE originally states. The problem, as we see it, is a loose competency definition. Thus, the necessary competency level before the trainee is allowed to progress to independent procedural performance is often defined in terms of subjective ratings. One author defines the prerequisite competence level as “practice repetitively until they felt comfortable” [100], and another uses experts’ procedural performances as benchmarks, creating a level that comes close to an actual ML [84].

In contrast, ML is defined by high-standard learning goals, reached by continuous formative feedback. In the studies by Barsuk [71, 72, 78, 88, 96, 99], Cohen [67] and colleagues, a four-hour course of dedicated simulation-based ML was used for practicing central venous catheterization. These studies adhere to the principles of ML as defined by Bloom in his original work by using pretesting and training with immediate feedback until a predefined mastery level is reached. The positive results in all Kirkpatrick/Phillips levels of these studies, as earlier discussed, indicates that a focus on high mastery standards and feedback even in short ML courses enables the transfer of skills training to clinical performance and patient care and is cost-efficient.

#### Control groups

The use of control groups adds credibility to the results of a study by controlling for external factors influencing the results. Only applying extra attention to an intervention group additionally introduces the risk of a Hawthorne effect [58]. This effect can be estimated by granting a control group attention by subjecting them to a different intervention within the same time period.

Although a randomized controlled design is not easily applicable to educational interventions due to difficulty in blinding and the risk of rub-off effects, 11 of the included studies have done so to some extent [69, 70, 76, 77, 80, 81, 85, 94, 95, 98, 101]. By random allocation to groups, the underlying characteristics of the groups are thought to be evenly distributed, thus diminishing the bias of inherent differences in trainees [102].

Instead of randomization, a historical group at the same institution is used for control [66–69, 71–75, 78, 79, 96, 97, 99], which is thought to imply that the physical settings were identical. However, the temporal separation of the two groups will likely introduce confounders, such as changes in procedural guidelines, new equipment or differences in patient characteristics. Attempting to bridge this difference, some papers report patient and trainee characteristics [74, 76] while also declaring differences in guidelines, practices or other confounders.

In addition to including a control group, the description of control group training is important for the evaluation of the effect of the study. Unfortunately, description detail of control group training varies widely in the studies. Exemplary control group descriptions are primarily from studies defining a control group receiving a different, but still novel, training regime [70, 73, 77, 94]. At the other end of the detail spectrum, studies describe the training received by controls as observing more experienced physicians before their own independent performances [68, 69, 85, 93].

In the ML studies by Barsuk, Evans, Cohen et al., the traditional training was five CVC insertions performed under supervision before the resident obtained the right to practice the procedure independently [67, 76, 78]. The intervention of a 4- to 5-h course with a high passing standard thus represents a significant shift in the assessment of competence before independent practice and could be a key reason for the positive results of these studies.

#### Measuring methods

The fact that only three studies investigate Kirkpatrick/Phillips level 5, return on investment, may be due to the time-consuming measurement, relying on valid clinical and economical information. Further, in keeping with the principles of Kirkpatrick/Phillips, only the interventions showing positive results in the lower levels of evaluation are eligible for higher-level evaluations [53, 55]. This hierarchy results in the selection of only positive results of lower level studies for further investigations of higher-level outcomes.

Level 4 effects are primarily reported as decreases in patient complications or surrogate measures of these, such as the number of needle passes. We would argue that the actual number of complications should be the gold standard, although the surrogate measures are strong predictors of risk of complications [78]. For both level 4 and 5 studies, several confounding factors such as guideline changes, introduction of novel equipment or a shift in patient categories could induce doubt of the causality of effect. That 11 studies report positive level 4 and 5 effects nevertheless provides an indication of CBE and ML-based CVC training as being beneficial to both patient outcome and creating a return on investment.

Studies describing Kirkpatrick level 3 use both checklists identical to the ones used in the preclinical simulation setting [77, 79, 98, 103] and specific checklists developed for the clinical setting [70, 76, 95, 98] to determine the transfer and retention of skills. The criticism of checklist usage for evaluation of competence has previously been mentioned [35].

Using the same checklists for the skills measurement of the inexperienced and the proficient competence level could fail to recognize the traits of the expert. Experts rely upon pattern recognition cultivated by years of experience rather than on rigid task flow charts of competency training and assessment [36, 103, 104]. Proficient performers may thus receive low scores or even fail an assessment made for basic competence assessment.

Dwyer et al. proposes a solution to this challenge by using a modified Angoff method [105] to determine passing scores for residents at different levels of expertise. The study demonstrates a high correlation between judges, suggesting uniformity in the expected level of competence [105]. The included studies by Barsuk, Cohen, Diederich et al. [67, 71, 72, 78, 88, 91, 96, 99] also used the Angoff method to determine the minimal passing score used to determine mastery, although only for one level.

Retention of skills over time plays an important part in training, benefitting the intended patients for a longer period. The interval for the evaluation of retention in the included studies is variable and ranges from 4 weeks to over 2 years after the completion of the educational intervention [91, 92]. Short retention intervals may be insufficient to capture competence decay over time, whereas long intervals increase the risk that confounding factors will influence the results. The results of the reviewed studies show a predominant decrease in skills over time.

### Strengths and limitations of the study design

This review suffers from four potential limitations.

First, it focuses solely on basic procedural anesthesia skills training for novice trainees. As such, the conclusion we draw is of the basic level of skills acquisition. This induces a risk of overlooking the higher-level learning in more advanced proficiency training. Widening the scope of this study to include the higher-level training of more senior doctors would most likely have introduced an even larger heterogeneity of the included studies, making conclusions even more difficult to assert.

Second, the limitation of using the Kirkpatrick/Phillips model is its risk of oversimplifying the causality of training effect. Even if establishing effects on all five levels, efforts should be made to declare all other factors to solidify the conclusions of causality. This declaration is rarely done in the reviewed studies and thus introduces a bias to our conclusions that cannot be estimated.

Third, this review could be criticized for the same infallibility discourse by not questioning the structural concepts of CBE, as stated by Boyd [32]. We used an outcomes-based evaluation method to evaluate a likewise outcomes-based training method, which could be seen as a non-critical appraisal of CBE. Although we agree with the necessity for a critical approach to the conceptual constructs of behavioristic learning theory, this more theoretical discourse would be better served in a separate review.

Fourth, the purpose of a narrative review is to review the literature for strengths and weaknesses, gaps and areas for consolidation but without calculating effect sizes. The limitation of such a review is inversely linked to the adequacy, breadth and depth of the literature search. In our search, we incorporated several relevant databases and searched the references of the selected literature for gray literature. We thus believe that we have made an adequate effort to include all available literature, thereby adding strength to our conclusions.

### Implications for clinical implementation

ML-based studies create the most consistent positive results in all Kirkpatrick/Phillips levels and thus appeal as the preferable learning strategy. As so many studies are investigating the same learning strategy and from the same study group, this would be stretching the conclusion a bit. The large heterogeneity of other studies, intervention, and assessment design adds to this caveat, making it difficult to systematically assess or calculate an aggregate effect of the studies. The often more rigorously defined mastery level together with continuous feedback could nevertheless be a way to achieve higher competence and thus counter the criticism of mediocrity.

When constructing CBE curricula, the medical educator must pay attention to the assessment methods. The Angoff method is a widely accepted method of standards setting [105, 106]. Using it to describe several levels of proficiency for the same competence or skill would further enable the continuous learning process and document the progress of the trainees.

The original Angoff method uses expert judges to determine an expected passing score for a level of proficiency [107]. In the modified version, multiple rounds of iterations are used to enhance agreement between the experts. Data from the resulting tests can then be used to further enhance the credibility of the passing score [108]. The Angoff method is thus not limited to determining the passing score of expected minimal competence but could be used for calculating scores for all levels of expertise [108]. Creating and using assessment standards for all expected competence levels would counter the criticism of promoting mediocrity and minimum standards.

Implementing novel training programs also requires careful planning. The description of the necessary efforts for the dissemination of a successful training program to a different setting from Barsuk spotlights the importance of an implementation strategy [72]. Identifying and securing the support of key players is vital in this process. If successful implementation is achieved, the trickle-down effect also from the same intervention holds the promise of an additional trade-off effect from the intervention [99].

### Future research directions

The evidence from the three included studies demonstrating return on investment seems to indicate a substantial economic gain from especially ML and to a lesser extent CBE. Future studies should aim to replicate these results as well as those in levels 1–5 in different settings and define control groups vigorously in order to establish generalizability. Furthermore, comparing different training interventions could generate additional knowledge of the most effective way of conducting CBE training.

Increasing residents’ contribution to clinical service could further add to the return on investment evaluation. Training in a more systematic way could enable earlier independent procedural performance while at the same time enhancing the quality and safety of the procedural performance. Thus, the gain from the intervention may be even greater than by decreased complications alone, providing further argument to medical educators looking for change.

Retention studies should aid in establishing an optimal interval for remedial training in order to maintain the originally learned skills. This could be achieved by sequential testing of residents at intervals after their initial training, determining when the skills decay results in subpar performance of the procedure. This time point would be variable, influenced by the procedure’s complexity, performance frequency and the severity of the consequences from subpar performance. Potentially lifesaving, complex and seldom-performed procedures would thus warrant shorter interval for remedial training to ensure the expected standard.

## Conclusion

It is a continuous challenge for educators and administrators to accommodate economical demands to train the best possible doctors within an acceptable time frame and at an acceptable cost. ML seems to satisfy both factors at the basic graduate anesthesia education level. High mastery level increases the competence level expected of the competent junior doctor while keeping in line with the outcome-focused CBE.

In conclusion, medical researchers evaluating the effects of CBE and ML in basic anesthesiology training should focus on both return on investment and patient-related outcomes in order to justify the enhanced supervision involved and cost of training. The evidence gained from future rigorous, controlled, stepwise educational evaluation studies would be a pivotal argument in favor of CBE and ML in the ongoing economic prioritization debate.

## Abbreviations

CBE: Competency-based education
CVC: Central venous catheterization
EDC: Epidural catheter
ML: Mastery learning
